# Therapeutic Potential of Selected Medicinal Plant Extracts against Multi-Drug Resistant *Salmonella enterica* serovar Typhi

**DOI:** 10.1016/j.sjbs.2021.10.008

**Published:** 2021-10-16

**Authors:** Sadaf Naz, Sadia Alam, Waseem Ahmed, Shah Masaud Khan, Abdul Qayyum, Maimoona Sabir, Alia Naz, Asia Iqbal, Yamin Bibi, Sobia Nisa, Amany Salah Khalifa, Amal F. Gharib, Ahmad El Askary

**Affiliations:** a Department of Microbiology, The University of Haripur, Haripur 22620, Pakistan; b Department of Horticulture, The University of Haripur, Haripur 22620, Pakistan; c Department of Agronomy, The University of Haripur, Haripur 22620, Pakistan; d Department of Environmental Sciences, The University of Haripur, Haripur 22620, Pakistan; e Department of Wildlife & Ecology, University of Veterinary and Animal Sciences, Lahore, Pakistan; f Department of Botany, PMAS-Arid Agriculture University Rawalpindi, Rawalpindi 46300, Pakistan; g Department of Clinical Pathology and Pharmaceutics, College of Pharmacy, Taif University, Taif 21944, Saudi Arabia; h Department of Clinical Laboratory Sciences, College of Applied Medical Sciences, Taif University, Taif 21944, Saudi Arabia

**Keywords:** *Adhatoda vasica*, *Amaranthus hybridus*, *Aloe barbadensis*, Phytochemical analysis

## Abstract

*Salmonella enterica* serovar Typhi is Gram negative, rod shaped, facultative anaerobic bacterium, belongs to enterobacteriaceae family that causes typhoid fever in humans. This bacterium has become a super bug due to acquisition of multi drug resistance. Bacteria is transmitted through food and water contaminated with human feaces. Present study reports the screening of *Adhatoda vasica*, *Amaranthus hybridus* and *Aloe barbadensis* and their evaluation against multi-drug resistant *Salmonella enterica* serovar Typhi. Qualitative analysis of ten phytochemicals was conducted using chemical method and Gas Chromatography-Mass Spectrometry (GCMS). Antibacterial activity of plants was carried out by agar well diffusion method on Mueller Hinton agar. Total tannins, total alkaloids and total flavonoids of different parts of three plants were estimated through spectrophotometer. Total tannins content in different parts of plants was present in the given order *Amaranthus hybridus* leaf > *Aloe barbadensis* leaf > *Adhatoda vasica* leaf > *Adhatoda vasica* flower > *Adhatoda vasica* stem. Whereas, the order of total flavonoid concentration was *Amaranthus hybridus* leaf > *Aloe barbadensis* leaf > *Adhatoda vasica* leaf > *Amaranthus hybridus* seed. Total alkaloids have order, *Adhatoda vasica* leaf > *Amaranthus hybridus* leaf > *Adhatoda vasica* flower > *Amaranthus hybridus* seed > *Aloe barbadensis* leaf. Results of phytochemical analysis suggested that plants have strong profile of antioxidants, total phenolic contents and various enzymes proposing them best alternate to cure bacterial infections. GC-MS analysis further confirmed stronger phytochemical profile that can be utilized as antagonists to *Salmonella enterica* serovar Typhi.

## 1 Introduction

Enteric fever is the most prevalent bacterial infection in developing countries caused by *Salmonella enterica* serovar Typhi. Bacteria are transmitted by the intake of faecal contaminated water or food (Newell et al., 2010). Headaches, chills, high fever, nausea and malaise are the characterized sign and symptoms of typhoid (Cock, 2008). Annually 12.2 million cases (Murray et al., 2012) and 190,200 deaths are reported worldwide, mostly amongst the children between the age group of 2–5 years (Lozano et al., 2012). Almost 30% of the bloodstream bacterial infections in Asia and 10% in Africa that arise in the general populations are caused by the etiological agent *Salmonella enterica* serovar Typhi (Deen et al., 2012). About 0.2 million deaths due to typhoid have been reported by WHO in 2014, most of them were reported from Asia (Andrews et al., 2017, WHO, 2017). Recent surveys conducted in Asian countries suggested 12–20 million cases of typhoid fever with 30% mortality rate, and if the condition remains untreated this rate may reach up to 90% (Mogasale et al., 2014, Obaro et al., 2017). Usually chloramphenicol, ampicillin, trimethoprim and sulfamethaxaole are the first line of therapy for the typhoid fever. As the cases of multi drug resistance (MDR) reported since 1970 and 1980, the efficiency of those drugs became uncertain (Klemm et al., 2018) alternatives were tested. At first, the second line of therapy regarding multi drug resistance fluoroquinolones was effective, but with passage of time resistance to floroquinolones has also been reported (Raveendran et al., 2008). Contrary to synthetic antibiotics medicinal plants are important antimicrobial resources to combat multidrug resistance. Currently, more than 80% of world population uses medicinal plants as a source of treatment against different ailments (Riaz et al., 2021). Isolation and validation of vibrant antimicrobial components from medicinal plants permits the production of new medicines. Thus, it is beneficial to explore the medicinal properties of plants collected from different sources (Mujaddidi et al., 2021). *Adhatoda vasica* is commonly known as Malabar nut, exhibit medicinal properties against various pathogens and therefore used in Ayurvedic medicines since long time (Maurya and Singh, 2010, Kaur et al., 2012). It is commonly found in Malaysia, India, Himalayan region, Sri Lanka and Burma (Kaur et al., 2012). Major components of a plant i.e. vasicinol, vasicine, vasicinolone, vasicol and adhatonine are mainly present in *Adhatoda vasica*. Various pharmacological properties i.e. antibacterial, anti-malarial, anti-cancerous and anti-inflammatory are exhibited by *Adhatoda vasica*.

*Amaranthus hybridus* is commonly known as “pigweed”, having height of 1–6 feet. This plant has light green color, hairy and rough leaves, having small flower and pink or red color taproot (Mepha et al., 2007). Various solvent extracts of *Amaranthus* are used in Thai, Chinese and Indian medicines to cure various infections such as gynecological infections, diarrhea, UTI and respiratory tract infections. *A. hybridus* is also utilized to reduce pain and cure inflammation (Baral et al., 2011). All species of *Amaranthus* have various anti-inflammatory and antioxidant potentials. Saponins, alkaloids, terpenoids, phenolic acids, flavonoids, vitamins and amino acids have been evaluated from different parts of *Amaranthus* (Kumar et al., 2011, Nana et al., 2012, Sharma et al., 2012).

*Aloe barbadensis* has juicy leaves and is a stem less plant having length of 60–100 cm. *Aloe barbadensis* has thick leaves which are green in color while a number of plants show white spots on the lower and upper surface of the stem (Tyler, 1993). Water content of *Aloe barbadensis* gel is 99.3% while the other 0.7% consists of mannose and glucose. Leaf of *Aloe barbadensis* acts as skin care products due to the presence of these sugars in combinations with amino acids and other enzymes (Agarry et al, 2005). Peptic ulcer and other gastrointestinal infections can be cured by using *Aloe barbadensis* gel (Thiruppathi et al., 2010, Johnson et al., 2011). Significant antibacterial, antioxidant, anti-inflammatory and antifungal activities of *Aloe vera* gel are reported in various studies (Fani and Kohanteb, 2012, Nejatzadeh-Barandozi, 2013, Baradaran et al., 2013, Ray et al., 2013, Kang et al., 2014, Vijayalakshmi et al., 2012, Sitara et al., 2011). Antitumor and anti-aging activity of the plant is also reported while it also has applications in treatment of cardiac disorders (Chatterjee et al., 2013). *Aloe vera* consists of almost 100 phytochemical components for that reason it plays a vital role in herbal medicines since time immemorial.

The typhoidal pathogen has become resistant to different generations of commercially available drugs. Also the role of selected medicinal plants against multi drug resistant *Salmonella enterica* serovar Typhi is inadequate. So the current study was designed to evaluate the phytochemical profile of various solvent extracts from different parts of selected medicinal plants and to evaluate their antibacterial activities against the multidrug resistant typhoidal bacterium.

## 2 Material and methods

The study was designed to evaluate the phytochemical screening and antibacterial activity of three different indigenous medicinal plants against multi drug resistant *Salmonella enterica* serovar *Typhi* strains and its respective reference strain.

### 2.1 Plants collection

*Adhatoda vasica, Amaranthus hybridus* and *Aloe barbadensis* were selected for current study and collected from The University of Haripur and different localities of Haripur region, Khyber Pakhtunkhwa, Pakistan from July to September 2020. The selected plants are commonly available in District Haripur. The voucher specimen of each plant has been submitted to Department of Horticulture, The University of Haripur for future reference.

#### 2.1.1 Pre-extraction of plant samples for Soxhlet’s extraction

For extraction, healthy and disease-free plants were collected and washed with normal tap water to remove dust and other impurities and then washed with distilled water to remove different microbes present on plants surface. The plants materials were shade dried by following the guideline used by Sasidharan et al. (2011) to avoid direct contact with heat and light to prevent denaturation of light sensitive constituents of plants. Then these plants were ground to fine powder, mixtures were made by mixing 50 g plants powder with 100 ml solvent (methanol, ethyl-acetate, hexane and chloroform respectively) and then added to Soxhlet’sApparatus (Behr Labor- Technik.Germany-2013) as described by Ncube, (2008). The cyclic process was continued until final product was obtained. Drying of extracts were processed in freeze drier at temperature of −60 to −65 °C for 24 h.

### 2.2 Screening for qualitative analysis

#### 2.2.1 Sample preparation

For sample preparation, 5 ml distilled water, 10 ml hydrogen chloride and 2 ml of plant extract were taken in a test tube and then filtered. These filtrates were further used for phytochemical screening.

#### 2.2.2 Alkaloids detection

All the extracts were individually dissolved in HCl and mixture was filtered. Mayer’s test was used for alkaloid's detection. Potassium mercuric iodide (1 ml) was added to 2 ml of individual extract and formation of yellow colored precipitate indicated the presence of alkaloids.

#### 2.2.3 Carbohydrates detection

All the extracts were mixed with 5 ml distilled water and filtered. Aqueous extracts were further processed for carbohydrates detection by Molisch’s test. 2 ml extract was taken in test tube and 2–3 drops of alcoholic α-naphthol solution was added. Violet ring formation indicated the presence of carbohydrates.

#### 2.2.4 Glycosides detection

Diluted hydrochloric acid was added to 2 ml of each extract and Borntrager’s test was used with slight modifications. Ferric chloride solution (2–3 drops) was added to 2 ml of individual extract and kept in water bath for 5 min. The solution was cooled and then benzene was added v/v to that extract. Layer of benzene was removed, and ammonia solution was added to the solution. Presence of glycoside was detected by the formation of rose-pink color in ammonical layer.

#### 2.2.5 Saponin detection

Froth test was used for detection of saponins in plant extract for which 10 ml of distilled water was added to 2 ml of extract and shaken for 10–15 min. Presence of saponins was detected by formation of 1 cm foam layer.

#### 2.2.6 Phytosterol detection

Salkowski’s Test was performed for the detection of phytosterol in plant extract. Chloroform (1 ml) was added to 2 ml extract and then filtered. 2 ml of conc. sulphuric acid was added to the filtrate and left to stand for few minutes and presence of phytosterols was detected by the formation of golden yellow color.

#### 2.2.7 Phenol detection

For phenol detection ferric chloride test was performed. Ferric chloride solution of 3–4 drops was added to 2 ml of extract. Presence of phenols was detected by the formation of bluish black color.

#### 2.2.8 Tannins detection

In plant extract, presence of tannins was detected by using Gelatin test. Individual extract of about 2 ml were treated with 2 ml of 1% gelatin solution. Presences of tannins were detected by the formation of white precipitates.

#### 2.2.9 Flavonoids detection

Alkaline reagent test was applied for flavonoid detection for which sodium hydroxide solution (2 ml) was added to 2 ml extract. Presence of flavonoids was detected by the appearance of intense yellow color.

#### 2.2.10 Detection of proteins

Xanthoproteic test was performed, which is one of the common tests for the detection of total proteins in the plant extract. Nitric acid (2–3 drops) was added to extract in test tube and proteins were detected by the appearance of yellow color.

#### 2.2.11 Detection of diterpenes

Copper acetate test was applied for detection of diterpenes. Copper acetate (2–3 drops) was added drop by drop to 2 ml of extract, presence of diterpenes was detected by appearance of green color (Obasi et al., 2010).

### 2.3 Quantitative analysis

#### 2.3.1 Sample preparation for total phenolic and antioxidants detection

Folin-Ciocalteau reagent was used to detect total phenolic content. Extraction mixture was prepared by the ratio 90:8:2 i.e. 90 ml of methanol was mixed with 8 ml acetone and 2 ml hydrochloric acid. Then 2 ml of plant sample were mixed with 20 ml of digestive mixture. The sample was vortexed, centrifuged at 11000 rpm for 15 min and the supernatant was collected in eppendorf tubes and further processed for total phenols. The residues were dissolved in 5 ml distilled water in test tubes to make different concentration i.e. 0.2, 0.4, 0.6 µg/500 µl. FC reagent was introduced to each tube and left for about 3 min. Then 20% Na_2_CO_3_ (2 ml) was added to each tube and mixtures were mixed vigorously. The tubes were kept in water bath for about 1 min, cooled down and absorbance was measured at 765 nm wavelength. By taking different concentrations of gallic acid, standard curve was prepared. Absorbance of each sample was measured in triplicate and then final value was taken in µg of gallic acid equivalent (Srinivasan and Kumaravel, 2015).

#### 2.3.2 Antioxidants detection

Different concentrations of extracts i.e. 50, 100, 150 µg/ml were used for antioxidants detection. DPPH (1, 1-diphenyl 1–2-picrylhydrazyl) 0.004%, was prepared in 80% methanol solution. 5 ml of DPPH solution was added to each tube and incubated for 30 min and absorbance was measured at 517 nm wavelength using spectrophotometer (UV/VIS T80+). The total scavenging activity was measured by the ratio of absorption of the sample to the control (DPPH 0.1 mM was taken as control).

Absorbance of antioxidant was calculated as following,

Radical scavenging activity (%) = (Control – Sample) / Control × 100.

#### 2.3.3 Total alkaloid content

Plant extract of 1 g was mixed with 20% H_2_SO_4_ and 20 ml ethanol by ratio of 1:1. Mixture was filtered and 1 ml of filtrate was mixed with 60% H_2_SO_4_ (5 ml). Mixture was left for 5 min and after that 5 ml of 0.5% formaldehyde was added to the mixture and left for 3 h. The absorbance was measured at 565 nm (Ekwueme et al., 2015).

#### 2.3.4 Total flavonoid

Total flavonoid content was determined by Kim et al., (2003). Plant extract (1 g) was taken in a tube and 4 ml of distilled water was added to it. Aluminum chloride solution (10%) i.e. 0.3 ml was added to it. For 5 min tubes were incubated at 27 °C and 2 ml of NaOH was introduced to the test tubes and then about 1–2 ml of distilled water was added to the mixture and the tubes were vortexed. Absorbance was measured at 510 nm by the appearance of pink color. All the values were taken in triplicate.

#### 2.3.5 Total tannins content

By following Folin and Ciocalteu (FC) method tannins were quantified. Plant extract of 0.5 ml was mixed with 3.75 ml distilled water, 0.5 ml of 35% sodium carbonate solution and 0.25 ml FC reagent was added to it, and the absorbance was measured at 725 nm (Puneetha et al., 2014).

#### 2.3.6 Samples preparation for enzymes detection

Plant extracts of 1 g/ml were mixed with 2 ml phosphate buffer (pH 7–7.8). Mixture was vortexed and then centrifuged for 3 min at 11000 rpm, supernatant was collected in Eppendorf tubes for further enzymes quantification.

#### 2.3.7 Peroxidase (POX)

Phosphate Buffer (pH 5) 100 µl, 100 µl H_2_O_2_ (40 Mm) and 100 µl of guaicol was added to 100 µl of reaction mixture (prepared from methanol, acetone and HCl by the ratio of 80:9:2). Then 100 ul of prepared sample i.e. enzyme extract was introduced to the mixture. Absorbance was measured at wavelength of 47 0 nm. Absorbance of each sample was measured in triplicate and then final value calculated using formula,

μg /gram = (Control – Sample) / Control × 100

#### 2.3.8 Superoxidase dismutase (SOD)

Phosphate buffer 500 µl, methionine 200 µl, Triton X 200 µl and nitro blue tertazolium (NBT) 100 µl was added to 100 µl enzyme extract. 800 µl distilled water was added to the mixture. The mixture was kept in UV light for about 15 min and then 100 µl riboflavin was added. Absorbance was taken at the wavelength of 560 nm at spectrophotometer. Absorbance of each sample was measured in triplicate and then final value was calculated using formula,

μg /gram = (Control – Sample) / Control × 100

#### 2.3.9 Catalase (CAT)

Enzyme extract of 100 µl by the method mention above was mixed with 100 µl H_2_O_2_. Absorbance was measured at 240 nm wavelength. Absorbance of each sample was measured in triplicate and then final value was taken as follows,

μg /gram = (Control – Sample) / Control × 100

### 2.4 Preparation of culture media for *Salmonella enterica* serovar typhi

Salmonella-Shigella agar (63 g/1000 ml) is a selective medium used for isolation and revival of Salmonella culture. Medium was autoclaved at 121 °C for 20 min and poured into sterile disposable Petri plates.

### 2.5 Preparation of Muller Hinton agar (MHA)

Muller Hinton Agar is commonly used for antibiotics sensitivity testing (38 g/l). Medium was autoclaved for 20 min at 121 °C, poured into petri plates and allowed to solidify.

### 2.6 Antibacterial activity

The antibacterial activity of selected plants was detected through agar well diffusion method. Clinical isolate of MDR *Salmonella enterica* serovar Typhi (SS1) was obtained from Pathology lab of District Head Quarter Hospital Haripur. This bacterium was resistant to ampicillin, gentamicin, ciprofloxacin, ceftriaxone, streptomycin and erythromycin. This strain was used as reference strain. Ertapenem was used as control and the strain was sensitive to ertapenem. Clinical isolate was spread on the surface of the Mueller Hinton agar plate. With the help of sterilized cork borer about 6–8 mm bores were made. Then different volumes of plants extracts i.e. 25 µl, 50 µl and 75 µl were introduced into the wells and Petri-plates were incubated for 24 h at 37 °C. After 24 h,’ zones of inhibition of each extract was measured in millimeter (Valgas et al., 2007).

### 2.7 Determination of minimal inhibitory concentration (MIC)

Minimum inhibition concentration of plants extracts was determined by using sterilized 96-well plates (Wiegand et al., 2008). To each well of 12 rows of plates, 125 µl sterilized nutrient broth was introduced. After that an extra 125 µl mixture of plant extract and nutrient broth was introduced from well 2 to 12 by making serial dilution of 40 mg/ml to 0.078 mg/ml. After that 5 µl of *Salmonella typhi* culture was introduced to each well from row 3 to 12, whereas row 1 was considered as negative control and row 2 was considered as positive control. Plates were incubated at 37 ^°^C for 24 h. Absorbance was measured at wavelength of 600 nm. Each value was taken in triplicate and minimum inhibition concentration of plant extract was calculated (Nisa et al., 2020).

### 2.8 Gas Chromatography-Mass Spectrometry (GCMS)

Plant extracts exhibiting promising antibacterial activity were selected for GC-MS profiling. Plant samples with good MIC value were further analyzed for quantitative phytochemical analysis using “Thermo Scientific (DSQII) GC”. The GC was equipped with a TR-5MS capillary column of length 30 M, Fill Thickness 0.25 μm and Internal Diameter of 0.25 mm. The carrier gas Helium (He) was used with flow rate of 1 ml/min. The injector was operated in split mode with temperature of 250 °C.

The sample volume 1 μl was injected with initial Oven temperature of 50 °C and held for 2 min, then increased to 150 °C with the temp rate of 8 °C/min and further increased to 300 °C with temperature rate of 15 °C/min and hold for 5 min.

### 2.9 Statistical analysis

Statistical analysis tool of MS-excel 2016 was used for the authentications of triplicate values of inhibition zones diameter and concentration values. Each experimental value was expressed in means and standard deviation (SD) was also calculated.

## 3 Results

### 3.1 Qualitative analysis of phytochemicals

**Fig 1:**
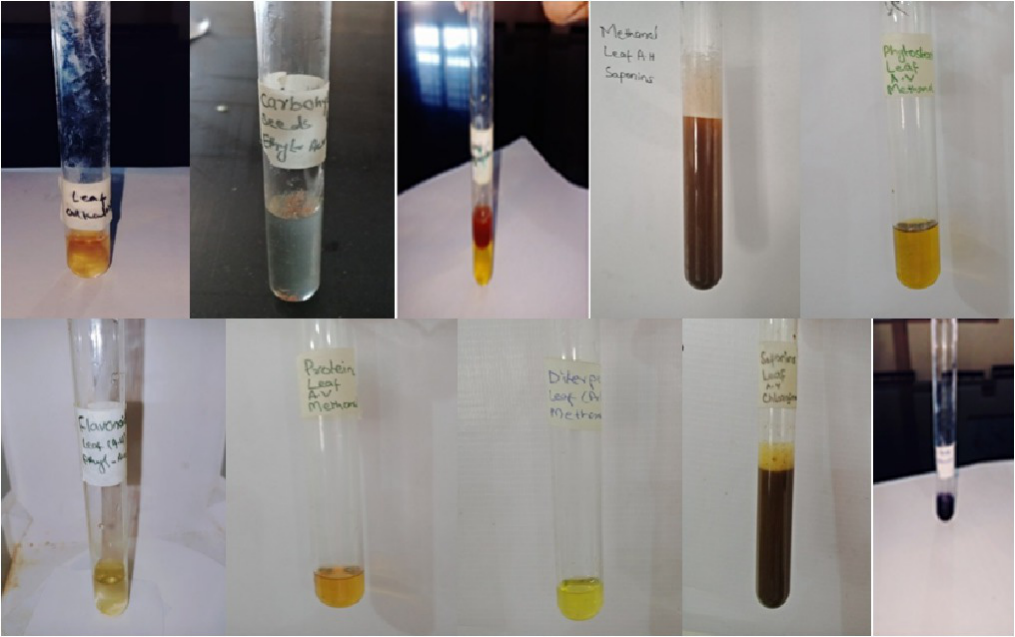
(a) Alkaloids in leaf of Adhatoda vasica, (b) Carbohydrates in seeds of Amaranthus hybridus in ethyl-acetate, (c) Glycosides in leaf of Amaranthus hybridus, d) Saponin in leaf of Amaranthus hybridus in methanol, (e) Phytosterols in leaf of Adhatoda vasica in methanol, (f) Flavonoids in leaf extracts of Adhatoda vasica in ethyl-acetate, (g) Protein detection in leaf of Adhatoda vasica in methanol, (h) Diterpenes in leaf of Adhatoda vasica in methanol, (i) Saponin in leaf of Adhatoda vasica in chloroform, (j) Phenols in leaf of Amaranthus hybridus.

**Fig. 2:**
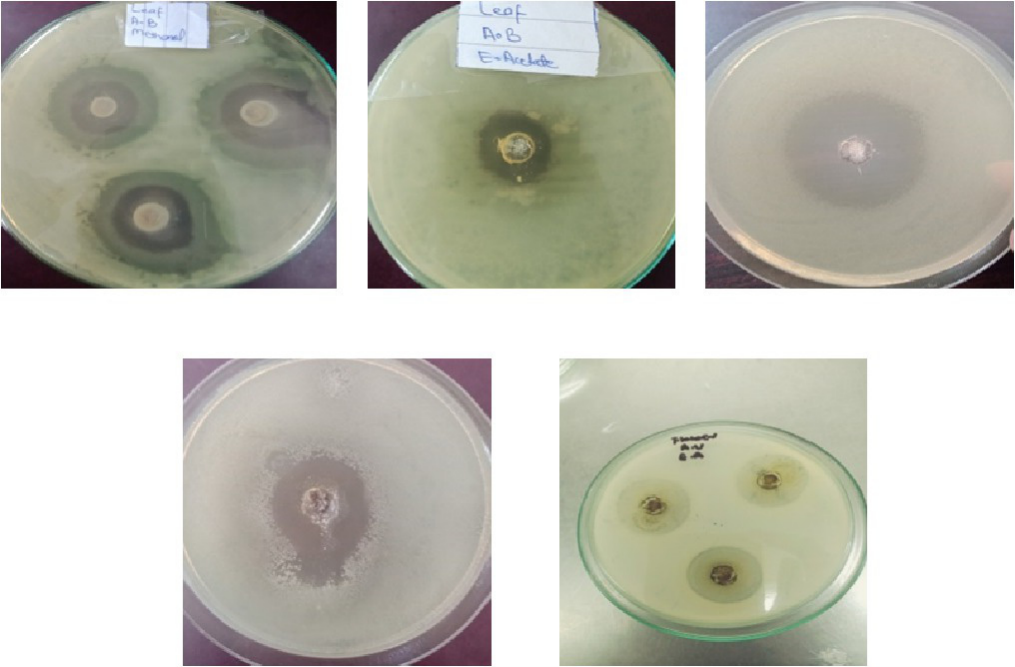
Zone of inhibition in mm of (a) leaf of *Aloe barbadensis* in methanol, (b) leaf of *Aloe barbadensis* in ethyl-acetate, (c) Leaf of *Adhatoda vasica* in methanol, (d) Leaf of *Amaranthus hybridus* in chloroform, (e) Flower *Adhatoda vasica* in ethyl-acetate.

The presence of alkaloids, phenols, diterpene, carbohydrate, proteins, phytosterol, tannins, flavonoids, glycosides, saponins in the leaf, seed stem and roots of *Amaranthus hybridus* was detected in various solvents (Fig. 1). The highest amount of carbohydrate was detected in leaf portion in solvent hexane, phytosterols and saponins were also detected in maximum amount in the leaf of *Amaranthus hybridus*. Alkaloids, tannins and saponins were not detected in *Amaranthus hybridus* (Table 1). Very high content of alkaloids was detected in the leaf and stem extract of *Adhatoda vasica* in different solvents (Table 2). Diterpenes, carbohydrates, tannins and saponins were also detected in the highest amount in the foliar part of *Adhatoda vasica.* All other bioactive components were present in moderate amount, while phenol and glycosides were totally absent in the root part of selected plant. Bioactive components in leaf and root extract of *Aloe barbadensis* were detected in various solvents (Table 3). The highest amount of carbohydrates, proteins, phytosterols and saponins were detected in the foliar part of *Aloe barbadensis.* No trace of diterpenes was detected in roots while all other components were present in moderate amount (Fig. 2).

**Table 1:** Bioactive components of *Amaranthus hybridis* extract in various solvents.

**Bioactive components**	**Part plant**	**Methanol**	**Chloroform**	**Ethyl-acetate**	**Hexane**
Alkaloid	Leaf	**+**	**++**	**–**	**++**
	Seed	**+**	**+**	**+**	**+**
	Stem	**++**	**++**	**–**	**+**
	Root	**–**	**–**	**–**	**–**
Phenols	Leaf	**++**	**++**	**++**	**++**
	Seed	**++**	**++**	**++**	**+**
	Stem	**+**	**+**	**+**	**–**
	Root	**+**	**–**	**–**	**+**
Diterpenes	Leaf	**+**	**++**	**+**	**+**
	Seed	**–**	**–**	**–**	**–**
	Stem	**+**	**+**	**+**	**+**
	Root	**+**	**+**	**+**	**+**
Carbohydrates	Leaf	**++**	**++**	**++**	+++*
	Seed	**++**	**++**	**+**	**++**
	Stem	**++**	**++**	**++**	**++**
	Root	**+**	**+**	**+**	**+**
Proteins	Leaf	**++**	**++**	**++**	**++**
	Seed	**++**	**++**	**+**	**+**
	Stem	**++**	**+**	**+**	**–**
	Root	**+**	**+**	**+**	**+**
Phytosterols	Leaf	+++*	+++*	**++**	+++*
	Seed	**+**	**+**	**+**	**+**
	Stem	**+**	**+**	**+**	**+**
	Root	**+**	**+**	**+**	**+**
Tannins	Leaf	**+**	**+**	**+**	**++**
	Seed	**+**	**+**	**–**	**–**
	Stem	**+**	**+**	**+**	**–**
	Root	**–**	**–**	**–**	**–**
Flavonoid	Leaf	**++**	**++**	**+**	**+**
	Seed	**++**	**++**	**+**	**++**
	Stem	**+**	**+**	**+**	**+**
	Root	**+**	**+**	**+**	**+**
Glycosides	Leaf	**++**	**++**	**++**	**++**
	Seed	**++**	**++**	**+**	**+**
	Stem	**+**	**+**	**+**	**+**
	Root	**++**	**+**	**++**	**+**
Saponins	Leaf	+++*	+++*	**++**	**++**
	Seed	**++**	**++**	**++**	**++**
	Stem	**++**	**++**	**+**	**++**
	Root	**–**	**–**	**–**	**–**

* Represent highest amount of bioactive components.

**Table 2:** Bioactive components of *Adhatoda vasica* in various solvents.

**Bioactive components**	**Plant part**	**Methanol**	**Chloroform**	**Ethyl-acetate**	**Hexane**
Alkaloid	Leaf	+++*	+++*	+++*	**++**
	Stem	**++**	**++**	+++*	**++**
	Flower	**+**	**+**	**+**	**+**
	Root	**+**	**+**	**–**	**–**
Phenols	Leaf	**++**	**++**	**++**	**++**
	Stem	**+**	**+**	**++**	**+**
	Flower	**+**	**+**	**+**	**+**
	Root	**–**	**–**	**–**	**–**
Diterpenes	Leaf	+++*	**++**	+++*	**++**
	Stem	**++**	**++**	**+**	**+**
	Flower	**++**	**++**	**++**	**++**
	Root	**+**	**+**	**–**	**–**
Carbohydrates	Leaf	**++**	+++*	**++**	**++**
	Stem	+++*	**++**	**++**	**++**
	Flower	**+**	**+**	**+**	**+**
	Root	**+**	**+**	**+**	**+**
Proteins	Leaf	**++**	**++**	**++**	**++**
	Stem	**+**	**+**	**+**	**+**
	Flower	**++**	**++**	**+**	**++**
	Root	**+**	**+**	**+**	**+**
Phytosterols	Leaf	**++**	**++**	**+**	**++**
	Stem	**++**	**+**	**+**	**+**
	Flower	**+**	**+**	**+**	**+**
	Root	**+**	**+**	**+**	**+**
Tannins	Leaf	+++*	+++*	**++**	**++**
	Stem	**+**	**+**	**+**	**+**
	Flower	**+**	**+**	**+**	**+**
	Root	**+**	**+**	**+**	**+**
Flavonoid	Leaf	**++**	**++**	**++**	**++**
	Stem	**++**	**+**	**++**	**++**
	Flower	**++**	**++**	**+**	**++**
	Root	**+**	**+**	**+**	**+**
Glycosides	Leaf	**++**	**++**	**+**	**+**
	Stem	**+**	**+**	**+**	**+**
	Flower	**++**	**++**	**+**	**+**
	Root	**–**	**–**	**–**	**–**
Saponins	Leaf	+++*	+++*	+++*	+++*
	Stem	**++**	**++**	**++**	**++**
	Flower	**++**	**++**	**++**	**++**
	Root	**++**	**+**	**+**	**+**

* Represent highest amount of bioactive components.

**Table 3:** Bioactive components in leaf and root extract of *Aloe barbadensis* in various solvent.

**Bioactive components**	**Plant part**	**Methanol**	**Chloroform**	**Ethyl-acetate**	**Hexane**
Alkaloid	Leaf	**++**	**++**	**++**	**++**
	Root	**+**	**+**	**+**	**+**
Phenols	Leaf	**++**	**++**	**++**	**++**
	Root	**++**	**++**	**++**	**++**
Diterpenes	Leaf	**++**	**++**	**++**	**++**
	Root	**–**	**–**	**–**	**–**
Carbohydrates	Leaf	+++*	+++*	+++*	**++**
	Root	**++**	**++**	**++**	**++**
Proteins	Leaf	+++*	**++**	**++**	**++**
	Root	**++**	**++**	**++**	**++**
Phytosterols	Leaf	+++*	+++*	+++*	**++**
	Root	**++**	**++**	**++**	**++**
Tannins	Leaf	**++**	**+**	**+**	**++**
	Root	**++**	**++**	**++**	**++**
Flavonoid	Leaf	**++**	**+**	**++**	**++**
	Root	**+**	**+**	**+**	**+**
Glycosides	Leaf	**++**	**+**	**+**	**+**
	Root	**++**	**+**	**+**	**+**
Saponins	Leaf	+++*	**++**	**++**	**++**
	Root	**++**	**++**	**+**	**+**

* Represent highest amount of bioactive components.

### 3.2 Quantitative analysis of phytochemicals

#### 3.2.1 Total alkaloid content determination

Leaf of *Adhatoda vasica* shows higher alkaloids content i.e. 9741.6 mg/100 g, which is followed by flower having 2816.8 mg/100 g. The leaf of *Amaranthus hybridus* had higher alkaloids content 5961.77 mg/100 g, while the alkaloids content of *Aloe barbadensis* leaf and roots was 1184.3 mg/100 g and 933.39 mg/100 g respectively (Table 6).

**Table 4:** Total phenolic and Antioxidants determination in *Amaranthus hybridus*, *Adhatoda vasica* and *Aloe barbadensis.*

**Plant extract**	**Part of plant**	**Total phenolic content TPC (mg/100 g)**	**% inhibition of DPPH**
*Adhatoda vasica*	Leaf	22.41	57.75
	Flower	18.32	47.34
	Stem	14.68	45.752
	Root	15.41	35.655
*Amaranthus hybridus*	Leaf	36.50	55.478
	Stem	15.05	44.186
	Seed	7.41	45.752
	Root	11.86	35.655
*Aloe barbadensis*	Leaf	39.23	55.478
	Root	34.68	44.186

**Table 5:** Enzymatic content of plants extracts, catalase, peroxidase, superoxidase dismutase in μg/g.

**Name of plant**	**Part of plant**	**Super oxidase dismutase (SOD)**	**Catalase (CAT)**	**Peroxidase (POX)**
*Adhatoda vasica*	Leaves	2523.06	631.7	1217.5
	Stem	1372.55	631.7	840.55
	Flower	1876.13	631.7	1217.5
	Roots	771.96	631.7	833.32
*Amaranthus hybridus*	Leaf	5505	631.7	2067.54
	Stem	1722.13	632.11	1195.3
	Seeds	1149.8	631.7	1460.38
	Roots	738.89	643.5	987.81
*Aloe barbadensis*	Leaf	4975.83	643.9	1767.04
	Roots	1048.4	643.9	964.4

**Table 6:** Total flavonoids, tannins and alkaloid contents of *Adhatoda vasica, Amaranthus hybridus* and *Aloe barbadensis* in mg/100 g.

**Name of plant**	**Part of plant**	**Total alkaloids**	**Total tanninss**	**Total flavonoid**
*Adhatoda vasica*	Leaves	9741.6	928.7	3092
	Stem	722.5	853.71	663.12
	Flower	2816.5	883.54	1056.5
	Roots	537.5	497.26	581.05
*Amaranthus hybridis*	Leaf	5961.77	6344.03	3906.3
	Stem	1492.5	813.01	1092.5
	Seeds	1917.1	851.25	1379.9
	Roots	658.5	634.2	577.58
*Aloe barbadensis*	Leaf	1184.3	1499.73	3593.19
	Roots	933.39	787.94	762.77

#### 3.2.2 Total flavonoid determination

The total flavonoid content in the leaf of *Adhatoda vasica* was 3092 mg/100 g which was higher as compared to the other parts of the plant. The total flavonoid content of stem, flower and roots was 663.12 mg/100 g, 1056.5 mg/100 g and 581.05 mg/100 g. The leaf of *Amaranthus hybridus* had 3906.3 mg/100 g total flavonoid content, while stem, seeds and roots of that plant harboured 1092.5 mg/100 g, 1379.9 mg/100 g and 577.58 mg/100 g flavonoids. The total flavonoids content of leaf of *Aloe barbadensis* was 3593.1958 mg/100 g which was much higher than the roots flavonoids content i.e. 762.7758 mg/100 g (Table 6).

#### 3.2.3 Total tannins determination

Foliar part of *Adhatoda vasica* showed higher tannins content i. e., 928.7 mg/100 g, which is followed by flower having 883.54 mg/100 g. The leaves of *Amaranthus hybridus* has higher tannins content 6344.03 mg/100 g, while the tannins content of *Aloe barbadensis* leaves and roots is 1499.73 mg/100 g and 787.94 mg/100 g sown in Table 6.

#### 3.2.4 Total phenolic compounds (TPC)

Higher phenolic content was measured in the *Amaranthus hybridus*, *Aloe barbadensis* and *Adhatoda vasica* leaves, flowers of *Adhatoda vasica*, seeds of *Amaranthus hybridus* and roots of *Aloe barbadensis* (Table 4).

### 3.3 Antioxidants

Percentage free radical scavenging activity was determined to detect antioxidant activity. Maximum activity was observed in foliar parts of *Amaranthus hybridus* followed by *Adhatoda vasica* and *Aloe barbadensis* with 57.75 and 55.478% radical scavenging potential. *Adhatoda vasica flower extract exhibited* 47.34 activity followed by seeds of *Amaranthus hybridus* where 45.75% inhibition of DPPH scavenging activity was noted (Table 4).

#### 3.3.1 Superoxide dismutase (SOD)

Leaf of *Adhatoda vasica* had 2523.06 μg/g SODs content, while in leaf of *Amaranthus hybridus* 5505 μg/g and was in leaves of *Aloe barbadensis* 4975.83 μg/g amount of SOD was measured (Table 5).

#### 3.3.2 Catalase (CAT)

The Catalase content of *Adhatoda vasica* and *Amaranthus hybridus* leaves was 631.7 μg/g while *Aloe barbadensis* leaves had 643.9 μg/g catalase content. The seeds of *Amaranthus hybridus* harboured 631.7 μg/g catalase content (Table 5).

#### 3.3.3 Peroxidase (POX)

*Adhatoda vasica* leaves and flowers had 1217.5 μg/g peroxidase content while *Amaranthus hybridus* leaves were having 2067.54 μg/g peroxidase followed by leaves of *Aloe barbadensis* with 1767.04 μg/g (Table 5).

### 3.4 Antibacterial activity of selected plants extract

Higher antibacterial potential was exhibited by *Adhatoda vasica* and *Amaranthus hybridus* leafy part extracts with 8 mm and 10.5 mm zones of inhibition against *S. enterica* serovar Typhi. Methanolic extract of *Aloe barbadensis* leaves showed 4.5 mm inhibition zone against the same pathogen (Table 7). Maximum zones of inhibition have been detected against *Salmonella typhi* by leaf extract of *Aloe barbadensis, Amaranthus hybridus* and *Adhatoda vasica* (Table 8).

**Table 7:** Zone of inhibition of MDR strain of *Salmonella enterica* serovar Typhi against *Amaranthushybridus, Adhatoda vasica and Aloe barbadensis*extracts measured in mm.

**Plant extract**	**Part**	**Methanol** (zone of inhibition in mm)			**Ethyl Acetate** (zone of inhibition in mm)			**Hexane** (zone of inhibition in mm)			**Chloroform** (zone of inhibition in mm)		
		25 μl	50 μl	75 μl	25 μl	50 μl	75 μl	25 μl	50 Μl	75 μl	25 μl	50 μl	75 μl
*Amaranthus hybridus*	Leaf	3	5	6	3	4	5	3	5	6.5	3.5	5	8
	Seed	1.5	2.5	3.5	1	3	3.5	2	3.5	5	3	4	4.5
	Stem	1	2	2.5	1	1.5	2	1	2	2	1.5	2	3.5
	Root	–	–	–	–	–	–	–	–	–	–	–	–
*Adhatoda vasica*	Leaf	6	7	9.5	5	6.5	8	5	7	11	5.5	6	10.5
	Flower	3	4	6	2	3.5	4.5	3	4	5.5	2	3.5	5.5
	Stem	1	1.5	2	1	2	2	2	2.5	3	1	2	2.5
	Root	–	–	1.5	–	–	–	–	1	1.5	–	–	–
*Aloe barbadensis*	Leaf	1.5	3	4.5	2	3.5	4	2	4	5.5	2.5	3	4.5
	Root	–	1	1	–	–	1	–	–	1	–	1	1

**Table 8:** Measurement of Minimum inhibition concentration (MIC) values in%

Name of Plants	40 mg/ml	20 mg/ml	10 mg/ml	5 mg/ml	2.5 mg/ml	1.25 mg/ml	0.625 mg/ml
Leaf AB Methanol	66%	87.02%	92.03%	90.80%	79.10%	60.38%	56.60%
Seed AH	77.0%	50%	78.50%	81.30%	87.73%	93.70%	87.29%
Leaf AB EA	64%	85.64%	83%	90%	41.70%	88%	86.80%
Leaf AH Methanol	81.6%	75.80%	87.60%	67.80%	81.70%	87.80%	83.30%
Flower EA	85.3%	74.66%	72.29%	86.10%	87.20%	89.40%	88.60%
Leaf AV Chloroform	80.5%	62.90%	37.70%	22.80%	36.10%	68.88%	63.16%
Stem AV Chloroform	87.4%	52.88%	54.91%	70.54%	93.20%	81.35%	62.71%
Root AV Methanol	76.5%	81.30%	76.70%	31.50%	70.10%	52.60%	53.80%
Leaf AV M	80.4%	72.80%	81.18%	83.92%	47.32%	74.80%	72.80%
Flower AV M	77.8%	78.20%	63.35%	74.65%	77.55%	69.92%	66.70%
Leaf AH H	70.9%	75.66%	71.75%	79.45%	69.60%	68.40%	69.24%
Leaf AB M	87.6%	82.80%	86.80%	67.92%	59.70%	54.60%	55.30%

### 3.5 Minimum inhibition concentration (MIC)

A dose dependent response was observed for different concentrations of extracts. The highest inhibition was recorded for *Adhatoda vasica* leaf i.e. 92.03% at 10 mg/ml concentration, while the seeds extract of *Amaranthus hybridus* in hexane showed the inhibition percentage 93.70% at 1.2 5 mg/ml. Chloroform extract of *Adhatoda vasica* eleaf xhibited the highest inhibition at 40 mg/ml, hexane derived leaf extract of *Amaranthus hybridus* showed 79.45% inhibition at 5 mg/ml.

### 3.6 Gas Chromatography-Mass Spectrometry (GCMS)

The GC-MS analysis of *Amaranthus hybridus*, *Adhatoda vasica* and *Aloe barbadensis* revealed the existence of bioactive compounds. The identified chemical profile of *Amaranthus hybridus* indicated 41 compounds (Table9). The GC-MS results indicated 57 different compounds of *Adhatoda vasica* (Table10). Bioactive compounds identified from the hexane extract of *Aloe barbadensis* leaf contained 17 active compounds (Table 11). The bioactive compounds identification was established on the basis of the peak area, and retention time (Table 9, Table 10, Table 11).

**Table 9:** Bioactive compounds identified from the methanol extract of *Amaranthus hybridus* leaf.

**S. No**	**Name of Compounds**	**Formula**	**RT**	**Peak Area**	**Peak Height**
1.	Sarreroside	C_30_H_42_O_1_	0.76	29410903.95	1163646.41
2.	Cyclopropanedodecanoic acid, 2-octyl-, methyl ester	C_24_H_46_O_2_	1.51	23970480.01	911492.53
3.	13-Heptadecyn-1-ol	C_17_H_32_O	2.13	20218650.65	585802.71
4.	2Hydroxyethylphosphine	C_2_H_7_OP	3.30	49226951.70	3268477.27
5.	Digitoxin	C_41_H_64_O_1_	3.81	6172972.22	522580.09
6.	Butanoic acid, 4-hydroxy	C_4_H_8_O_3_	4.30	12165080.19	918952.08
7.	1,8-Di(4-nitrophenylmethyl)-3,6-diazaho moadamantan-9-one	C_23_H_24_N_4_O_5_	4.75	18390815.93	835304.00
8.	Mequinol	C_7_H_8_O_2_	5.73	17552561.95	576147.83
9.	*tert*-Hexadecanethiol	C_16_H_34_S	6.26	0189735.02	10189735.02
10.	Aspidospermidin-17-ol, 1-acetyl-19,21-epoxy-15,16-dimethoxy	C_23_H_30_N_2_O_5_	6.96	3063737.80	131237.99
11.	2-Oxazolamine, 4,5-dihydro-5-(phenoxymethyl)-N-[(phenylamino)carbonyl]	C_17_H_17_N_3_O_3_	8.05	41206391.30	1136190.72
12.	2-Methoxy-4-vinylphenol	C_9_H_10_O_2_	9.26	347743553.41	23156333.16
13.	2-Butanone, 4-(2,6,6-trimethyl-1,3-cyclohexadien-1yl	C_13_H_20_O	11.07	23825996.29	1042982.48
14.	1-Heptatriacotanol	C_37_H_76_O	11.56	5276619.78	401356.29
15.	2-Methyl-4-(2,6,6-trimethylcyclohex-1-e nyl)but-2-en-1-ol	C_14_H_24_O	11.84	3401973.26	392620.46
16.	Ingol 12-acetate	C_22_H_32_O_7_	12.11	1882844.57	263061.14
17.	Ppropiolic acid, 3-(1-hydroxy-2-isopropyl-5-methylcycl ohexyl)	C_13_H_20_O_3_	12.32	1395907.09	210863.52
18.	2-Benzothiazol	C_7_H_5_NS	12.81	154365701.11	4178962.77
19.	Megastigmatrienone	C_13_H_18_O	13.91	42798392.47	3415260.52
20.	Ingol 12-acetate	C_22_H_32_O_7_	14.75	9088216.46	461050.35
21.	9,10-Secocholesta-5,7,10(19)-triene-1,3-diol, 25-[(trimethylsilyl)oxy]-, (3á,5Z,7E)	C_30_H_52_O_3_Si	16.07	11608140.61	590332.43
22.	4-((1E)-3-Hydroxy-1-propenyl)-2-methoxyphenol	C_10_H_12_O_3_	16.54	76392171.46	3000155.81
23.	N-(2-Methylbutyl)(2E,4E,8Z,10E)dodecatetraenamide	C_17_H_27_NO	17.44	3395822.46	321986.34
24.	7,10-Epoxy-6H-azepino[1,2-e]purine-8, 9-diol, 4-amino-7,8,9,10-tetrahydro-, [7R-(7à,8à,9à,10à)]	C_10_H_11_N_5_O_3_	17.84	2935573.24	172575.59
25.	Hexadecanoic acid, 1-(hydroxymethyl)-1,2-ethanediyl ester	C_35_H_68_O_5_	18.25	3395572.86	358239.87
26.	n-Hexadecanoic acid	C_16_H_32_O_2_	18.74	24476899.75	1827030.15
27.	1-Propyl-3,6-diazahomoadamantan-9-ol	C_12_H_22_N_2_O	19.13	51577033.30	2519739.73
28.	Phytol	C_20_H_40_O	19.74	256768220.13	18146480.82
29.	1-Heptatriacotanol	C_37_H_76_O	21.02	5179464.52	397899.07
30.	2,2,4-Trimethyl-3-(3,8,12,16-tetramethyl-heptadeca-3,7,11,15-tetraenyl)-cyclohexanol	C_30_H_52_O	21.47	20681755.34	931829.38
31.	Hexadecanoic acid,2,3-dihydroxypropyl ester	C_19_H_38_O_4_	22.10	30006466.07	1278935.05
32.	E,E,Z-1,3,12-Nonadecatriene-5,14-diol	C_19_H_34_O_2_	23.04	44169437.34	2259393.52
33.	Cholestan-3-ol, 2-methylene-, (3á,5à)	C_28_H_48_O	23.51	16145255.46	861081.47
34.	3,3a-Epoxydicyclopenta[a,d]cyclooctan-4á-ol, 9,10a-dimethyl-6-methylene-3á-isopropyl	C_20_H_32_O_2_	23.87	10420422.96	687228.04
35.	9,12,15Octadecatrienoic acid, 2,3-bis[(trimethylsilyl)oxy]propyl ester, (Z,Z,Z)	C_27_H_52_O_4_Si_2_	24.65	5361621.25	221080.45
36.	Cholestan-3-one, cyclic 1,2-ethanediyl aetal, (5á)	C_29_H_50_O_2_	25.03	5288845.30	517531.7
37.	Stigmasterol	C_29_H_48_O	25.86	22339759.39	1057363.41
38.	Acetic acid17-acetoxy-4,4,10,13-tetramethyl-7-oxo −2,3,4,7,8,9,10,11,12,13,14,15,16,17-tet radecahydro-1H-cyclopenta[a]phenanth ren-3-yl (ester)	C_25_H_36_O_5_	26.59	512237.92	73780.06
39.	Acetic acid, 17-acetoxy-4,4,10,13-tetramethyl-7-oxo −2,3,4,7,8,9,10,11,12,13,14,15,16,17-tet radecahydro-1H-cyclopenta[a]phenanth ren-3-yl (ester)	C_25_H_36_O_5_	26.94	944673.01	88089.79
40.	Methyl 3á-hydroxyolean-18-en-28-oate	C_31_H_50_O_3_	27.35	3537093.97	332475.21
41.	Prosta-5,13-dien-1-oic acid, 9,11,15-tris[(trimethylsilyl)oxy]-, trimethylsilyl ester, (5Z,9à,11à,13E,15S)	C_32_H_66_O_5_Si_4_	27.80	394114.81	48016.64

**Table 10:** Bioactive compounds identified from the methanol extract of *Adhatoda vasica* leaf.

**S.No**	**Compounds Name**	**Formula**	**Peak Area**	**Peak Height**	**RT**
1.	Sarreroside	C_30_H_42_O_10_	29410903.9	1163646.41	0.76
2.	Cyclopropanedodecanoic acid, 2-octyl-, methyl ester	C_24_H_46_O_2_	23970480.0	911492.53	1.51
3.	13-Heptadecyn-1-ol	C_17_H_32_O	20218650.6	585802.71	2.13
4.	2Hydroxyethylphosphine	C_2_H_7O_P	49226951.7	3268477.27	3.30
5.	Digitoxin	C_41_H_64_O_13_	6172972.22	522580.09	3.81
6.	Butanoic acid, 4-hydroxy	C_4_H_8_O_3_	12165080.1	918952.08	4.30
7.	1,8-Di(4-nitrophenylmethyl)-3,6-diazaho	C_23_H_24_N_4_O_5_	18390815.9	835304.00	4.75
8.	Mequinol	C_7_H_8_O_2_	17552561.9	576147.83	5.73
9.	tert-Hexadecanethiol	C_16_H_34_S	10189735.0	360913.17	6.26
10.	Aspidospermidi-17-ol 1-acetyl-19,21-epoxy-15,16-dimethoxy-	C_23_H_30_N_2_O_5_	3063737.8	131237.99	6.96
11.	2-Oxazolamine,	C_17_H_17_N_3_O_3_	41206391.30	1136190.72	8.05
12.	2-Methoxy-4-vinylphenol	C_9_H_10_O_2_	347743553.41	23156333.16	9.26
13.	2-Butanone	C_13_H_20_O	23825996.29	1042982.48	11.07
14.	1-Heptatriacotanol	C_37_H_76_O	5276619.78	401356.29	11.56
15.	2-Methyl-4-(2,6,6-trimethylcyclohex-1-e -enyl)but-2-en-1-ol	C_14_H_24_O,	3401973.26	392620.46	11.84
16.	Ingol 12-acetate	C_22_H_32_O_7_	1882844.57	263061.14	12.11
17.	Propionic acid	C_13_H_20_O_3_	1395907.09	210863.52	12.32
18.	2-Benzothiazol	C_7_H_5_NS	154365701.11	4178962.77	12.81
19.	Megastigmatrienone	C_13_H_18_O	42798392.47	3415260.52	13.91
20.	Ingol 12-acetate	C_22_H_32_O_7_	9088216.46	461050.35	14.75
22.	9,10-Secocholesta-5,7,10(19)-triene-1,3 -diol, 25-[(trimethylsilyl)oxy]-, (3á,5Z,7E)-	C_30_H_52_O_3_Si	11608140.61	590332.43	16.07
23.	4-((1E)-3-Hydroxy-1-propenyl)- 2-methoxyphenol	C_10_H_12_O_3_	76392171.46	3000155.81	16.54
24.	N-(2-Methylbutyl)(2E,4E,8Z,10E)- dodecatetraenamide	C_17_H_27_NO	3395822.46	321986.34	17.44
25.	7,10-Epoxy-6H-azepino[1,2-e]purine-8,9 -diol, 4-amino-7,8,9,10-tetrahydro-, stereoisomer	C_10_H_11_N_5_O_3_	2935573.24	172575.59	17.84
26.	Hexadecanoic acid, 1-(hydroxymethyl)- 1,2-ethanediyl ester	C_35_H_68_O	3395572.86	358239.87	18.25
27.	n-Hexadecanoic acid	C_16_H_32_O_2_	24476899.75	1827030.15	18.74
28.	1-Propyl-3,6-diazahomoadamantan-9-ol	C_12_H_22_N_2_	51577033.30	2519739.73	19.13
29.	Phytol	C_20_H_40_O	256768220.13	18146480.82	19.74
30.	1-Heptatriacotanol	C_37_H_76_O	5179464.52	397899.07	0. 76
31.	2-Methyl-4-(2,6,6-trimethylcyclohex-1-e -enyl)but-2-en-1-ol	C_14_H_24_O,	3401973.26	392620.46	11.84
32.	Ingol 12-acetate	C_22_H_32_O_7_	1882844.57	263061.14	12.11
38.	9,10-Secocholesta-5,7,10(19)-triene-1,3 -diol, 25-[(trimethylsilyl)oxy]-, (3á,5Z,7E)-	C_30_H_52_O_3_Si	11608140.61	590332.43	16.07
39.	4-((1E)-3-Hydroxy-1-propenyl)- 2-methoxyphenol	C_10_H_12_O_3_	76392171.46	3000155.81	16.54
40.	N-(2-Methylbutyl)(2E,4E,8Z,10E)- dodecatetraenamide	C_17_H_27_NO	3395822.46	321986.34	17.44
41.	7,10-Epoxy-6H-azepino[1,2-e]purine-8,9 -diol, 4-amino-7,8,9,10-tetrahydro-, stereoisomer	C_10_H_11_N_5_O_3_	2935573.24	172575.59	17.84
42.	Hexadecanoic acid, 1-(hydroxymethyl)- 1,2-ethanediyl ester	C_35_H_68_O	3395572.86	358239.87	18.25
43.	n-Hexadecanoic acid	C_16_H_32_O_2_	24476899.75	1827030.15	18.74
44.	1-Propyl-3,6-diazahomoadamantan-9-ol	C_12_H_22_N_2_	51577033.30	2519739.73	19.13
45.	Phytol	C_20_H_40_O	256768220.13	18146480.82	19.74
46.	1-Heptatriacotanol	C_37_H_76_O	5179464.52	397899.07	0.76
47.	2,2,4-Trimethyl-3-(3,8,12,16-tetramethyl -heptadeca-3,7,11,15-tetraenyl)-cyclohexanol	C_30_H_52_O	20681755.34	931829.38	1.51
48.	Hexadecanoic acid, 2,3 -dihydroxypropyl ester, (ñ)-	C_19_H_38_O_4_	30006466.07	1278935.05	2.13
49.	E,E,Z-1,3,12-Nonadecatriene-5,14-diol	C_19_H_34_O_2_	44169437.34	2259393.52	3.30
50.	Cholestan-3-ol, 2-methylene-, (3á,5à)-	C_28_H_48_O	16145255.46	861081.47	3.81
51.	3,3a-Epoxydicyclopenta[a,d]cyclooctan -4á-ol, 9,10a-dimethyl-6-methylene -3á-isopropyl-	C_20_H_32_O_2_	10420422.96	687228.04	4.30
52.	9,12,15-Octadecatrienoic acid, 2,3-bis[(trimethylsilyl)oxy]propyl ester,	C_27_H_52_O_4_Si_2_	5361621.25	221080.45	4.75
53.	Cholestan-3-one, cyclic 1,2-ethanediyl aetal, (5á)-	C_29_H_50_O_2_	5288845.30	517531.76	5.73
54.	Stigmasterol	C_29_H_48_O	22339759.39	1057363.41	6.26
55.	Acetic acid,17-acetoxy-4,4,10,13-tetramethyl-7-oxo-2,3,4,7,8,9,10,11,12,13,14,15,16,17-tetradecahydro-1H- cyclopenta[a]phenanthren-3-yl (ester)	C_25_H_36_O_5_	512237.92	73780.06	6.96
56.	Methyl 3á-hydroxyolean-18-en-28-oate	C_31_H_50_O_3_	3537093.97	88089.79	8.05
57.	Prosta-5,13-dien-1-oic acid,	C_32_H_66_O_5_Si_4_	394114.81	332475.21	9.26

**Table 11:** Bioactive compounds identified from the hexane extract of *Aloe barbadensis* leaf.

**S.No**	**Compounds Name**	**Formula**	**RT**	**Peak Area**	**Peak Height**
1.	Trichloromethane	CHC_l3_	0.71	275763672.84	29867826.65
2.	Dimethylsulfoxoniumformylmethylide	C_4_H_8_O_2_S	4.71	167352.24	23367.82
3.	Fucoxanthin	C_42_H_58_O_6_	6.44	491143.24	24916.05
4.	Dimethyl Sulfoxide	C_2_H_6_OS	8.80	302328.71	26415.07
5.	Pregn-4-ene-3,20-dione,11,17,21-tris[(trimethylsilyl)oxy]-bis(O-methyloxime), (11á),	C_32_H_60_N_2_O_5_Si_3_	11.35	162316.00	16799.85
6.	4,25 Secoobscurinervan-4-one, O-acetyl-22-ethyl-15,16-dimethoxy-, (22à)	C_27_H_36_N_2_O_6_	13.93	861918.04	38379.41
7.	4,25-Secoobscurinervan-4-one, O-acetyl-22-ethyl-15,16-dimethoxy-, (22à)	C_27_H_36_N_2_O_6_	16.77	542567.25	21728.66
8.	Strychane, 1-acetyl-20à-hydroxy-16-methylene	C_21_H_26_N_2_O_2_	19.21	375351.55	14965.41
9.	Glycine, N-[(3à,5á,7à,12à)-24-oxo-3,7,12-tris[(trimethylsilyl)oxy]cholan-24-yl]-,methyl	C_36_H_69_NO_6_Si_3_	22.96	146669.62	18424.10
10.	Octasiloxane, 1,1,3,3,5,5,7,7,9,9,11,11,13,13,15,15-h exadecamethyl	C_16_H_50_O_7_Si_8_	25.41	52093221.24	1402009.46
11.	Hexasiloxane, 1,1,3,3,5,5,7,7,9,9,11,11-dodecamethyl	C_12_H_38_O_5_Si_6_	26.27	227024.26	30527.35
12.	Octasiloxane,1,1,3,3,5,5,7,7,9,9,11,11,13,13,15,15-h	C_16_H_50_O_7_Si_8_	27.09	1351954.70	40345.25
13.	Propanoic acid, 2-(3-acetoxy4,4,14-trimethylandrost-8en-17-yl)	C_27_H_42_O_4_	27.82	668153.09	52079.34
14.	Acetamide, N-[5-(diethylamino)-2-[(2,4-dinitrophen yl)azo]-4-methoxyphenyl]	C_19_H_22_N_6_O_6_	28.20	914441.12	60060.98
15.	Hexasiloxane,1,1,3,3,5,5,7,7,9,9,11,11-dodecamethyl	C_12_H_38_O_5_Si_6_	28.49	1247909.33	68276.81
16.	Octasiloxane,1,1,3,3,5,5,7,7,9,9,11,11,13,13,15,15-h exadecamethyl	C_16_H_50_O_7_Si_8_	29.47	770753.60	34580.94
17.	Octasiloxane,1,1,3,3,5,5,7,7,9,9,11,11,13,13,15,15-hexadecamethyl	C_16_H_50_O_7_Si_8_	29.90	402124.18	36104.36

## 4 Discussion

Plants have been used as a rich source of active compounds and preferred for the therapeutic purpose against number of diseases (Binish et al., 2021). Current study revealed some promising results of antibacterial activity of the selected plants against MDR typhoidal pathogen. Different species of *Amaranthus* have shown diverse antimicrobial activities. *Amaranthus viridis* chloroform foliar extracts exhibited activity against various microbes in a study conducted by Islam et al., (2010). *E. coli* showed greater sensitivity to alcoholic roots extracts of *Amaranthus hybridus*, while the same species root extracts in ethyl-acetates proved to be effective against *Staphylococcus aureus* (Dahiya et al., 2010). Wide range of antimicrobial activity of different species of *Amaranthus* i.e. *Amaranthus caudatus*, *Amaranthus hybridus* and *A. spinosis* foliar extracts in various solvents has been observed (Ahmed et al., 2013). Numerous microbes seem to be sensitive to leaf extracts of *Amaranthus hybridus* and this activity varies among various species at different concentrations. Leaf extracts of *Amaranthus hybridus* were found effective against *S. typhi, E. coli*, and *P. aeruginosae* having MIC range from 200 to 755 mg/ml (Maiyo et al., 2010).

Bioactivity of medicinal plants can be determined by the presence of different phytochemicals. Tannin, saponin, alkaloid, phenol, glycoside and flavonoids were detected in the leaf extract of *Aloe barbadensis* in a study conducted by (Ikpe, 2017) which is similar to our findings of various phytochemicals i.e. phenols, saponin, tannin, alkaloid, carbohydrate, glycosides and protein are present in different solvent extracts of *Aloe Barbadensis.* A study conducted by Maiyo et al., (2010) finds parallel results to our findings which revealed that *Amaranthus hybridus* can exhibit various phytochemicals such as terpenoids, flavonoids, glycosides and steroids having beneficial antimicrobial properties (Maiyo et al., 2010). Another study conducted by Betoni et al. (2006) on methanol and chloroform leaf extracts of *Adhatoda vasica* revealed similar results suggesting that these extracts contains tannins, glycosides, flavonoids and alkaloids contents.

A variety of organic actions have been acquired by *Adhatoda vasica* including anti-diabetic, anti-inflammatory, anti-jaundice, anti-microbial properties and also anti-spasmodic activity (Maurya and Singh, 2010). Different food borne pathogens are considered to be sensitive to *Adhatoda vasica* due to its antimicrobial potential (Subramaniam et al., 2015). Respiratory disorders i.e. cough, asthma, bronchitis and cold have been treated by the people since long time using *Adhatoda vasica* (Kaur et al., 2012). *Adhatoda vasica* shows strong antibacterial activity against various types of bacteria (Zabta et al., 2009). There are numerous biologically active constituents present in *Adhatoda* v*asica* that exhibited anti-bacterial activities. These components includes sterols, alkaloids, saponins, flavonoids and tannins which possess bactericidal potential against *Salmonella typhi* (Choudhury et al., 2013). Kumar et al., (2013) in his work stated that *Salmonella typhi* is more sensitive to methanol extract of *Adhatoda vasica* (Kumar et al., 2013).

Total phenolic content of methanolic leaf extract of *Aloe barbadensis* as determined by Bista et al. (2020) was 30.53 ± 0.30. Similarly, another study conducted by Kumar et al. (2011) revealed that phenolic content of 2.9 to 65.7 mg GAE per g of dry weight was present in *Aloe barbadensis* that is in accordance to our findings that TPC of leaf extract of *Aloe barbadensis* was 39.23 mg/100 mg. Antioxidant and phytochemical constituents of the medicinal plants vary due to the changed in different localities. By taking gallic acid as a standard, total phenolic content was measured and results shows that the leaf extracts of the *Adhatoda vasica* and *Amaranthus hybridus* in methanol showed higher content of phenols i.e. 22.41 mg/100 g and 36.50 mg/100 g which is in contrast to a study conducted by Klejdus et al. (2004). Their results showed higher phenolic contents in ethanol extract 279.25 ± 0.05 mg/g of leaf of *Adhatoda vasica* as compared to the methanol and hexane that is 89.28 ± 0.09 mg/g and 105.25 ± 1.05 mg/g. Another study conducted by Nana et al., (2012) showed phenolic content of 55–10.18 mgGAE/100 mg in leaf of *Amaranthus hybridus.* Concentration of total phenolic content of leaf extract of *Amaranthus hybridus* was detected 0.819 ± 0.0016 g GAE / 100 g dwb to 2.759 ± 0.0025 g GAE / 100 g dwb in a study conducted by Suffo et al. (2016), which was in contrast to our findings as our study detected 36.50 mg/100 g TPC in the leaf of *Amaranthus hybridus.*

Determination of alkaloids contents in polar solvent i.e. methanol is higher in the leaf of *Adhatoda vasica* 9741.6 mg/100 g, which is parallel to the results of another study showing stronger alkaloids contents in the polar solvents from the leaf of *Adhatoda vasica* 14.52 ± 0.26 mg/g (Klejdus et al., 2004). Total alkaloid content of leaf extract of *Aloe barbadensis* is 1184.3 mg/100 g, which is parallel to a study conducted by Iqbal and Ahmed (2021) as their study also shows higher content of alkaloid i.e. 1483.6 mg/g – 1670 mg/g in the leaf extract of *Aloe barbadensis.* Total flavonoid content of *Aloe barbadensis* leaf as determined by Iqbal and Ahmed (2021) varies from 0.53 mg/g −776.7 mg/g which are in contrast to our findings i.e 3593.19 mg/100 g.Total flavonoid content from leaf extract of *Adhatoda vasica* was 1550 mg QE/g in a study conducted by Kokati et al., (2013) while our study indicated total flavonoid content of leaf extract of *Adhatoda vasica* as 3092 mg/100 g.

The maximum DPPH radical scavenging activity of the methanolic leaf extracts of *Adhatoda vasica* was determined at a concentration of 200 µg/ml (Rachana et al., 2015). Our findings alsoindicated higher DPPH radical scavenging activity in polar solvent. Higher DPPH radical scavenging activity (105.33 µg/ml) was detected in methanolic extract of *Adhatoda vasica* leaves as reported by Paranthaman et al., (2012). Present study also indicates higher DPPH radical scavenging activity in foliar parts of all selected plants.

Medicinal plants possess different concentration of various enzymes having antimicrobial role (Brand, 2012). Superoxide dismutase (SOD) is considered as the plant defense enzyme as it plays anti-oxidative role in treating different plant diseases such as atherosclerosis and various other life threatening malfunctions. Higher concentration of Superoxide dismutase (SOD) have been reported in a study conducted by Brand, 2012, Brinda et al., 2013. Ahmed *et al*., (2018) also reported higher SOD content in the leaf extract of *Adhatoda vasica*. Our findings also manifest higher content of Superoxide dismutase (SOD) in the leaf extract of *Adhatoda vasica.*Catalase content of *Adhatoda vasica* leaf is also high i.e. 4629 µg/g followed by flower of *Adhatoda vasica* 2100 µg/g in a study conducted by Ahmed et al. (2018) which is parallel to our study outcomes. Peroxidase (POX) have been reported in higher content in leaf of *Adhatoda vasica* by Ahmed et al. (2018) and similar manifestations have been revealed in the current study.

Ramachandra et al. (2012) investigated the antibacterial activity of *Adhatoda vasica* against *Salmonella typhi* and found 17.50 mm, 13.16 mm and 11.50 mm zone of inhibition in methanol, hexane and chloroform extract respectively. Here, we also found methanolic extract more effective against *S. enterica* serovar Typhi. The trend of zone of inhibition was methanol > hexane > chloroform > ethyl-acetate. A study conducted by Lawrenceet al. (2009) revealed that *Salmonella typhi* showed various ranges of sensitivity against different solvent extract of *Aloe barbadensis* leaf and the maximum zone of inhibition was 9.66 mm in methanol. Methanolic extract of *Aloe barbadensis* leaf did not indicate promising results in our study. The difference may be attributed to different clinical isolate of *S. typhi* involved in both studies. Different concentrations of *Amaranthus hybridus* leaf extract against *Salmonella typhi* showed variable inhibition zones i.e. methanol extract (17.5 ± 2.0 mm at 100 µl/l), hexane extract (15.0 ± 1.4 mm at 50 µl/l), ethyl acetate (11.0 ± 1.7 mm at 100 µl/l) and 9.0 ± 1.4 mm at 50 µl/l (Maiyo et al., 2010). Matching results were obtained in present study and methanolic extracts showed higher zone of inhibition as compared to other solvents.Higher zone of inhibition was observed in hexane extract of *Adhatoda vasica* leaf followed by chloroform, methanol and ethyl-acetate. The MIC (0.125 mg/ml) of *Amaranthus hybridus* against *Salmonella typhi* (Chaudharyet al., 2017) was quite lower as compared to our findings (1.25 mg/ml).

On the basis of strong phytochemical profile against *S. typhi,* three samples were selected for GC-MS i.e. leaf of *Adhatoda vasica*, leaf of *Amaranthus hybridus* and leaf of *Aloe barbadensis*. GC-MS results of foliar part of *Adhatoda vasica* showed presence of various compounds identical to compounds reported in previous investigations (Srinivasan and Kumaravel, 2015). Similar compounds of both studies were phytol, 9,12,15- octadecatrienoic acid and hexadecanoic acid. These are bioactive compounds and play potential role against different microbes. GC-MS results of a study conducted by Suffo et al., 2016a, Suffo et al., 2016b indicated 18 different phytochemicals in the ethanol leaf extracts of *Amaranthus hybridus* which is in contrast to our findings of 42 different phytochemicals in the methanol leaf extracts of *Amaranthus hybridus*. Difference in solvents used for extraction and site of collection may influence the quantity. The bioactive compounds belong to different groups that were identified through GC-MS having various antimicrobial properties. Hexadacanonnic acid, phytol, cholestan, stigmasta, glycine and cyclopropaneoctanoic acid are frequently identified bioactive compounds and their antibacterial properties are already identified by various studies.

## 5 Conclusion

Evolution of antibiotic and multidrug resistance among pathogens are growing threat to human health worldwide. Self-medication and imbalance use of drugs has been developing resistance, and the presences of MDR infections including those of *Salmonella enterica* serovar Typhi that is resistant to many antibiotics of first and 2nd line of therapy including ciprofloxacin and ampicillin. Medicinal plants are the good source and an alternate to the resistant drugs. These contain higher concentration of antimicrobial agents such as tannins, alkaloids, flavonoids, phenolic, antioxidants and different enzymes which have the ability to degrade the oxygen reactive species through damage to their DNA, RNA and proteins. Current study has unravealed the detailed investigation about phytochemical compounds using GCMS against *S. typhi*. Further studies are recommended for isolation of novel more efficient antibacterial compounds against MDR for further clinical efficacy trials and easy and affordable testing.

## Declaration of Competing Interest

The authors declare that they have no known competing financial interests or personal relationships that could have appeared to influence the work reported in this paper.
